# Polyethylene Glycol Fusion and Nerve Repair Success: Practical Applications

**DOI:** 10.1016/j.jhsg.2024.01.016

**Published:** 2024-03-25

**Authors:** Benjamin A. Sarac, Matthew Wordsworth, Ryan W. Schmucker

**Affiliations:** ∗ Department of Plastic and Reconstructive Surgery, the Ohio State University Wexner Medical Center, Columbus, OH

**Keywords:** Axon, Fusion, Nerve, PEG, Wallerian

## Abstract

Peripheral nerve injuries are potentially devastating injuries leading to pain and impairment in motor and sensory functions. Since the first published use of microsurgical epineural repair of peripheral nerves in 1964, a wide variety of adjuncts have been studied. Polyethylene glycol is a fusogen that has been shown to restore axolemmal membranes. The use of polyethylene glycol in nerve injuries was first described in 1986, and animal studies have shown fusion of transected sensory and motor nerves following early application at the time of surgical repair with improved motor and sensory outcomes. Early human clinical trials have shown promising results, although more data are needed to provide specific indications and protocols. This article summarizes the background, current evidence, and future directions as well as potential applications of polyethylene glycol–mediated nerve fusion.

The motor, sensory, and pain sequalae of peripheral nerve injuries, regardless of etiology, can be potentially debilitating injuries for patients worldwide.^1^ The management and subsequent recovery of peripheral nerve injuries lies on the location, mechanism, and severity of the insult to the nerve.^2^ In 1964, Smith^3^ described the first microsurgical epineural repair, and the surgical management of a transected peripheral nerve that can be primarily repaired has changed little since this technique was described. Our understanding of the neurobiology of nerve repair and regeneration continues to improve, providing potential targets for intervention within the electrical, biochemical, and structural components of injured nerves.^4^ One technique gaining increased interest is the use of polyethylene glycol (PEG) to fuse axonal membranes at the time of surgical repair, with successfully fused axons resisting Wallerian degeneration (WD) at the site of injury and maintaining axolemmal continuity.^5^ Since its first discovery for nerve repair in 1986 by Bittner et al,^6^ PEG fusion has been studied in a range of animal models and human clinical trials. In this article, we discuss the mechanism of action, current evidence, and future directions for its potential use.

## Basic Science and Technique

Prior to understanding the mechanism of PEG fusion, it is first important to briefly review the peripheral nerve response to injury. In the case of a transected nerve (Seddon/Sunderland grade V—neurotmesis), the nerve begins to undergo WD, in which the nerve distal to the injury begins the active process of apoptosis.^7^^,^^8^ The calcium signaling that triggers WD begins at the time of injury, but the distal axons remain physically intact for 24–48 hours before axonal fragmentation.^9^ Once the distal axons have disintegrated, the debris is cleared by macrophages, and the local Schwann cells develop a regenerative phenotype to guide axonal regeneration through the surgical repair site down the now empty endoneurial tubes toward the distal targets.

Cellular fusion is a naturally occurring process and is used in basic science research for the creation of hybrid cells. Polyethylene glycol is a commonly used fusogen that lowers the activation energy required for membrane fusion; it is highly hydrophilic and thought to act by removing water from the phospholipid membranes, allowing adjacent membranes closely oppose and fuse.^9^ Polyethylene glycol with high and intermediate molecular weights is considered nontoxic and is used as an osmotic laxative medication in humans. In peripheral nerve injuries, PEG has been shown to restore axolemmal continuity applied to a repaired nerve prior to the onset of WD.^5^ The repair protocol in human studies closely resembles the protocol previously used in the study of PEG after transection of rat sciatic nerves.^10^

The technique of PEG fusion in peripheral nerve repair has been developed with three adjunct solutions to improve success rates of axonal fusion. Calcium ions signal both vesicular sealing of cut axons and subsequent WD. Therefore, a calcium-free solution is irrigated around the repair site prior to fusion. Methylene blue also prevents vesicle formation, which would seal the cut axons; therefore, a 1% solution is applied to both cut ends.^10^^,^^11^ This specific application of a calcium-free solution with an antioxidant, such as methylene blue, prior to application with PEG has been well-documented for many contemporary protocols and shown to improve fusion rates.^12^^,^^13^ The nerve ends must be freshly trimmed in the calcium-free environment as axons can begin to seal with 5 minutes.^11^ After microsurgical epineural repair, the PEG solution is applied to the coaptation site for 1–2 minutes before irrigating the area with a calcium-containing solution to wash away excess PEG and allow calcium-mediated sealing of any unfused axons. Figure 1, adapted from Neumann et al, provides a simplified version of the application process.^12^

**Figure:**
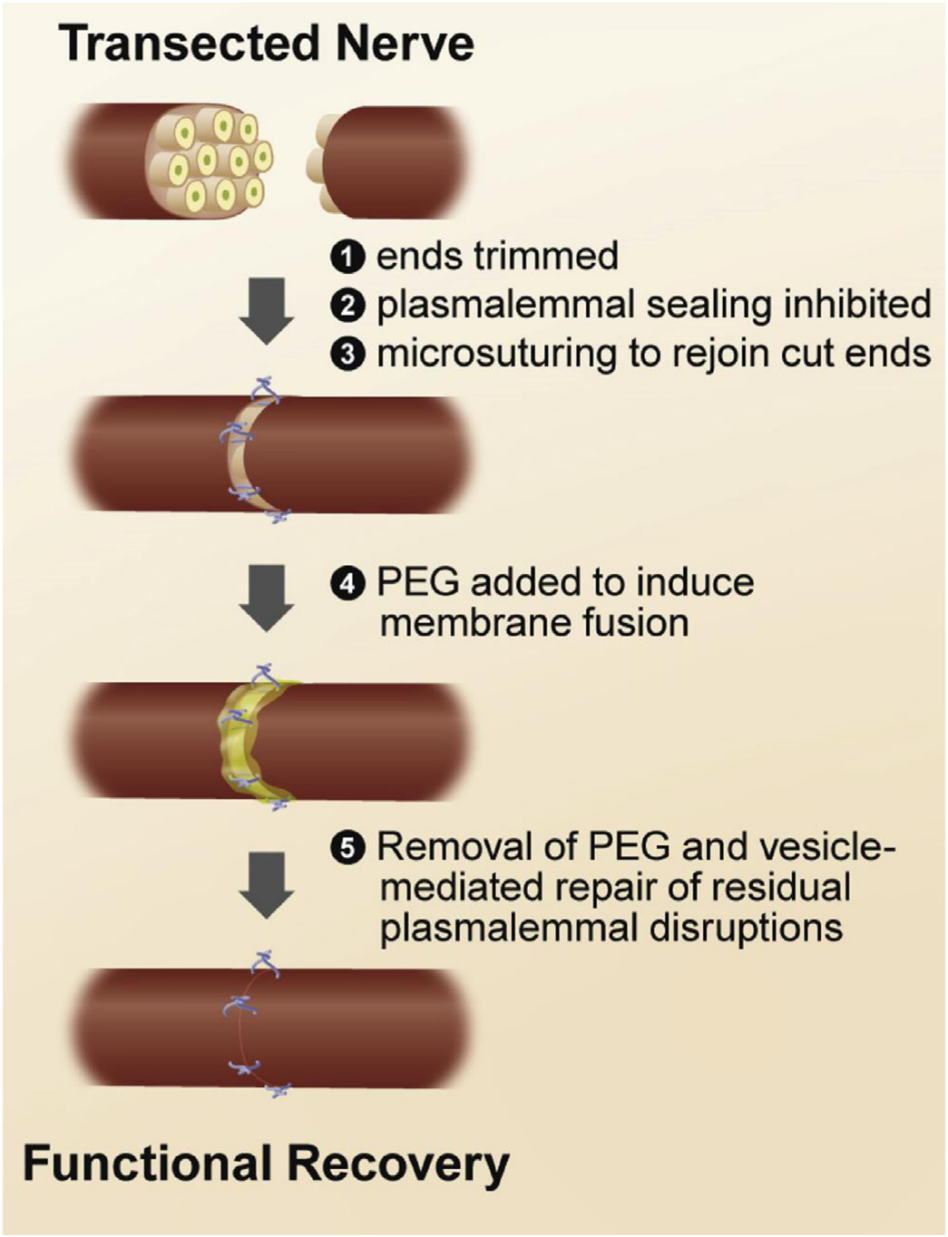
Repairing mammalian nerves with PEG-driven fusion. Figure adapted from Neumann et al.^12^

The effect of successfully fused axons is that axonal continuity across the coaptation site is restored within minutes. This restoration prevents WD in the successfully fused distal axons, and this axonal preservation prevents neuromuscular junction degeneration, thereby preventing target muscle atrophy. This in turn produces rapid and improved recovery of both motor and sensory functions. Nonfused axons will undergo WD and can subsequently regenerate through the “normal” process; however, they do not appear to be harmed by failure of fusion and PEG application.

A recognized potential downside of PEG fusion is that fusion is nonspecific. A proximal motor axon may fuse to a distal sensory axon or vice versa, and this would likely result in a nonfunctional axon. Studies have demonstrated this cross-innervation occurs, but redundancy, cortical plasticity, and axonal pruning appear to be sufficient to still improve functional outcomes compared with nonfused nerve repairs.^13^

## Human Trials

To date, there is limited evidence for the use of PEG fusion in humans, and only two reports are available in the literature. The first of this evidence was published in 2016 by Bamba et al,^14^ consisting of a case series of two patients undergoing repair of four digital nerve transections. Both patients had operative intervention within 12 hours of injury. They followed a standard protocol of resection and freshening of nerve endings, washing with a calcium-free solution, repairing with microsurgical technique, and treatment with methylene blue followed by application of PEG. Compared with control patients, the experimental arm with PEG application improved the sensory recovery (measured with monofilament testing and two-point discrimination) at 1, 4, and 8 weeks after surgery.

A second case report published by Lopez et al^15^ in 2022 is the only other published human trial using PEG fusion for peripheral nerves. They followed a similar protocol and included two patients. Patient one sustained a sensory-only digital nerve injury and, following repair, had subjective feeling in the fingertip 1–2 hours after surgery. By postoperative day 80, she had near-normal two-point discrimination. The second patient sustained a laceration of the median nerve at the level of the carpal tunnel, resulting in a mixed motor and sensory nerve injury. This patient was unfortunately lost to follow-up for some time but showed improved sensation over time and equivocal testing of his motor function, as preoperatively his abductor pollicis brevis was intact, but weak, despite the complete median nerve transection, suggesting dual innervation and confounding postoperative assessment.

These studies are limited by their sample size and generalizability, as it can often be difficult to perform digital nerve repair within 12–24 hours of injury owing to systematic constraints. The results, however, appear to be promising for continued study of PEG fusion of sensory nerves. Currently, there are at least five ongoing clinical trials evaluating the use of PEG fusion.^16^ The results of such studies may help provide further information on the indications for and specific protocols related to PEG fusion.

## Future Directions

The widespread adoption of PEG fusion is hampered by our understanding of its ideal use, the limited time window for acute intervention, and the technical challenges to achieve significant fusion. Animal models have focused on direct repair of cut or crush injuries with motor outcomes, and human trials have mostly reported on distal sensory nerves.^14^^,^^15^

The need to repair the nerve with PEG before the onset of WD will inevitability prevent ubiquitous use in traumatic nerve repair. There are known mouse phenotypes (Wld(S)) with delayed onset WD, and this discovery has enabled a better understanding of the mechanisms of WD. SARM1 is a key activation protein in WD as a NAD^+^ hydrolase; SARM1 inhibitors and NAD^+^ metabolism modulators are both molecular targets of interest in drug development for neurodegenerative diseases. If a drug that delays WD is approved for human use, this could change the window for successful primary repair of peripheral nerve trauma with PEG fusion. In animal studies, immunosuppression and limb cooling have both been shown to delay the onset of WD.^17^^,^^18^

A significant (yet largely unstudied) potential benefit of PEG fusion may be in more complex nerve transfer and nerve grafting surgery rather than in acute traumatic repair. For example, consider targeted muscle reinnervation, a procedure in which distal sensory nerve endings are coapted to expendable motor nerves for the prevention of neuroma formation and treatment of postamputation pain.^19^^,^^20^ The immediate partial restoration of axonal continuity in nerve transfers might avoid the early pain response that is often seen with targeted muscle reinnervation. Additionally, this technique, in theory, may permit faster rehabilitation after motor nerve transfers.

Polyethylene glycol fusion also holds promise in long segment nerve autografts or allografts. If successful fusion occurs at the proximal end of the graft and the distal end of the graft forms a second growth cone of successfully fused axons, this would in theory “jump” the regenerative distance of the graft for those fused axons. Ultimately this would allow restoration of innervation of targets that may otherwise have been too distal for reliable reinnervation within the time-critical window for motor endplates.

## Limitations

Although the promise of PEG fusion is exciting, it is not without its limitations, the first and most important of which is timing. Specifically in the setting of nerve transection, patients must undergo operative intervention prior to the initiation of WD. The specific window of use has not yet been established but is likely within the first week from injury and possibly even within the first 24–48 hours. Intervention with PEG application beyond this point may not provide the desired effects. Additionally, when treating with PEG fusion, it is known that the fusion is nonspecific. This means that a mixed motor and sensory nerve may have cross-fusion of the sensory and motor nerve endings after coaptation, thus preventing the restoration of normal anatomic alignment and function of the nerve.^13^

## Conflicts of Interest

Dr Schmucker participated in the Neuraptive Clinical Trial as the principal collaborator at our institution (Ohio State University) but received no direct individual funding neither the trial nor for this manuscript. No benefits in any form have been received or will be received by the other authors related directly to this article.
